# Emergency endovascular repair of blunt thoracic aortic injury-related pseudoaneurysm using a castor single-branched stent graft: a case report and literature review

**DOI:** 10.3389/fcvm.2026.1896389

**Published:** 2026-09-01

**Authors:** Yisong Wang, Haoyu Liu, Tonghe Zhang, Mingxuan Mao, Hongwei Zhang, Bo Chen, Xiaolin Tian, Yankui Li

**Affiliations:** 1 Department of Vascular Surgery, The Second Hospital of Tianjin Medical University, Tianjin, China; 2 Department of Rehabilitation Medicine, The Second Hospital of Tianjin Medical University, Tianjin, China; 3 Cardiovascular Center, The Second Hospital of Tianjin Medical University, Tianjin, China

**Keywords:** artery, blunt thoracic aortic injury, emergency, stent graft, thoracic aortic pseudoaneurysm

## Abstract

Blunt thoracic aortic injury (BTAI) is a severe traumatic condition caused by sudden deceleration and is associated with high mortality. When a complete aortic rupture does not occur, a thoracic aortic pseudoaneurysm (TAP) may develop, carrying a substantial risk of rupture and death. Thoracic endovascular aortic repair (TEVAR) has become the primary treatment modality for BTAI; however, its management remains challenging in patients with lesions adjacent to the left subclavian artery (LSA) and an insufficient proximal landing zone.

**Case report:**

A 38-year-old woman was admitted to the emergency department with precordial and shoulder-back pain that persisted for 2 h following a motor vehicle accident. Computed tomography angiography (CTA) revealed a pseudoaneurysm in the aortic isthmus accompanied by bilateral pleural effusion. Dynamic clinical observations demonstrated persistent pain, a progressive increase in pleural effusion volume, and a gradual decline in hemoglobin levels, suggesting unstable grade III BTAI with a high risk of rupture. Because the distance between the pseudoaneurysm entry tear and the LSA origin was less than 10 mm and the distance between the left common carotid artery (LCCA) and the LSA was approximately 7 mm, conventional TEVAR was unlikely to provide an adequate proximal landing zone. Emergency endovascular repair using a Castor single-branched stent-graft system was performed under general anesthesia. Intraoperative angiography demonstrated complete exclusion of the pseudoaneurysm, with preserved blood flow in the LSA and supra-aortic branch vessels. The postoperative course was uneventful. At the 3-year follow-up, CTA demonstrated satisfactory stent positioning without evidence of endoleak, stent migration, or branch occlusion as well as favorable aortic remodeling without recurrent visualization of the pseudoaneurysm. This case suggests that the Castor single-branched stent-graft may be a minimally invasive treatment option for patients with blunt thoracic aortic injury-related pseudoaneurysm involving or adjacent to the left subclavian artery, although further studies are required to confirm its safety and efficacy.

## 1 Introduction

Blunt thoracic aortic injury (BTAI) is a severe traumatic condition that most commonly occurs secondary to sudden deceleration trauma, particularly in road traffic accidents such as motor vehicle collisions. Although the incidence of this injury is extremely low, accounting for less than 1% of all traffic-related accidents, it is associated with a critical clinical course and an exceptionally high mortality rate. Once aortic rupture occurs, the mortality rate may reach 100%. BTAI-related deaths account for approximately 16% of all fatalities resulting from traffic accidents (1, 2). In cases where the aortic injury does not progress to complete rupture, a thoracic aortic pseudoaneurysm (TAP) may develop. The TAP is primarily composed of three components: a saccular aneurysmal cavity, an external fibrous pseudocapsule, and an entry tear in the aortic wall (3). Owing to the marked heterogeneity in clinical manifestations, patients presenting with typical symptoms such as chest pain or interscapular pain are more readily diagnosed, whereas patients with occult or nonspecific symptoms are prone to misdiagnosis or missed diagnosis (4). Therefore, an early definitive diagnosis and timely intervention are crucial for improving patient outcomes. Surgical repair was initially considered the principal treatment strategy for this condition. However, with advances in endovascular techniques, thoracic endovascular aortic repair (TEVAR) has enabled the rapid management of aortic injuries and has emerged as a less invasive therapeutic option (5). Herein, we report a case of thoracic aortic pseudoaneurysm caused by BTAI following motor vehicle collision-related deceleration trauma, which was successfully treated with emergency endovascular repair using a Castor single-branched stent graft and review the relevant literature.

## 2 Case report

### 2.1 Patient presentation

**Figure 1:**
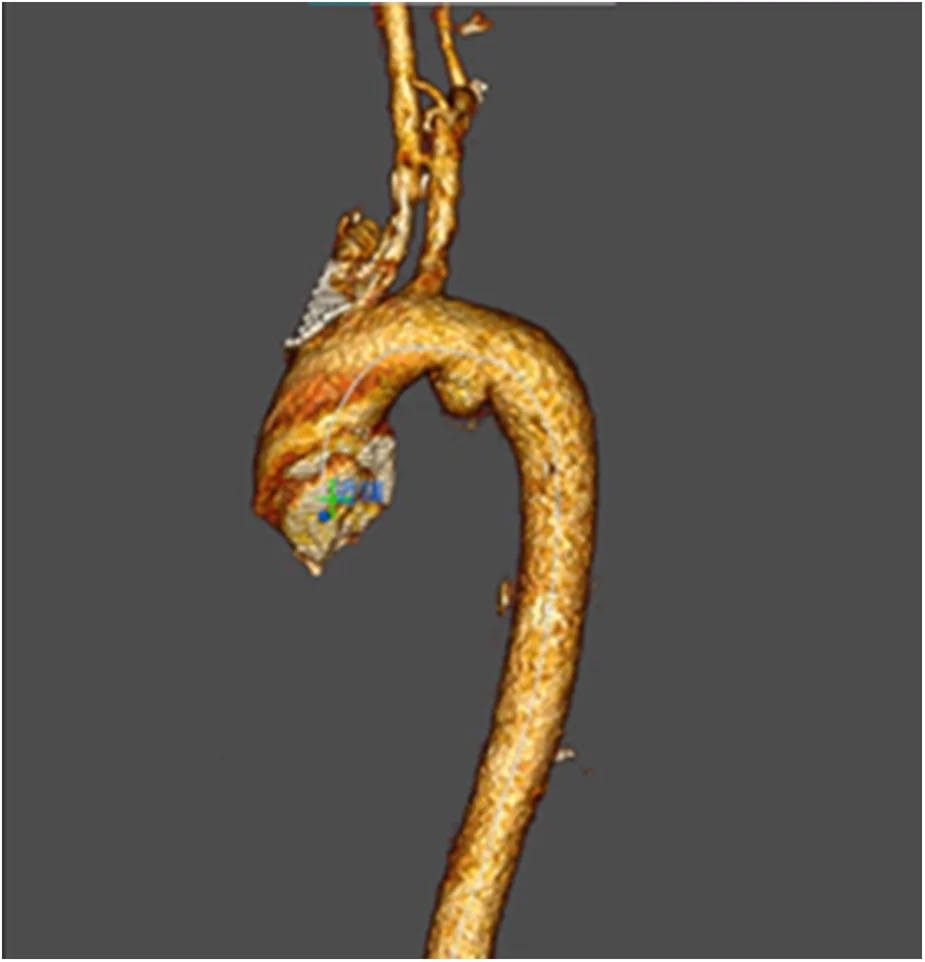
Thoracic aortic CTA demonstrating a pseudoaneurysm at the aortic isthmus.

**Figure 2:**
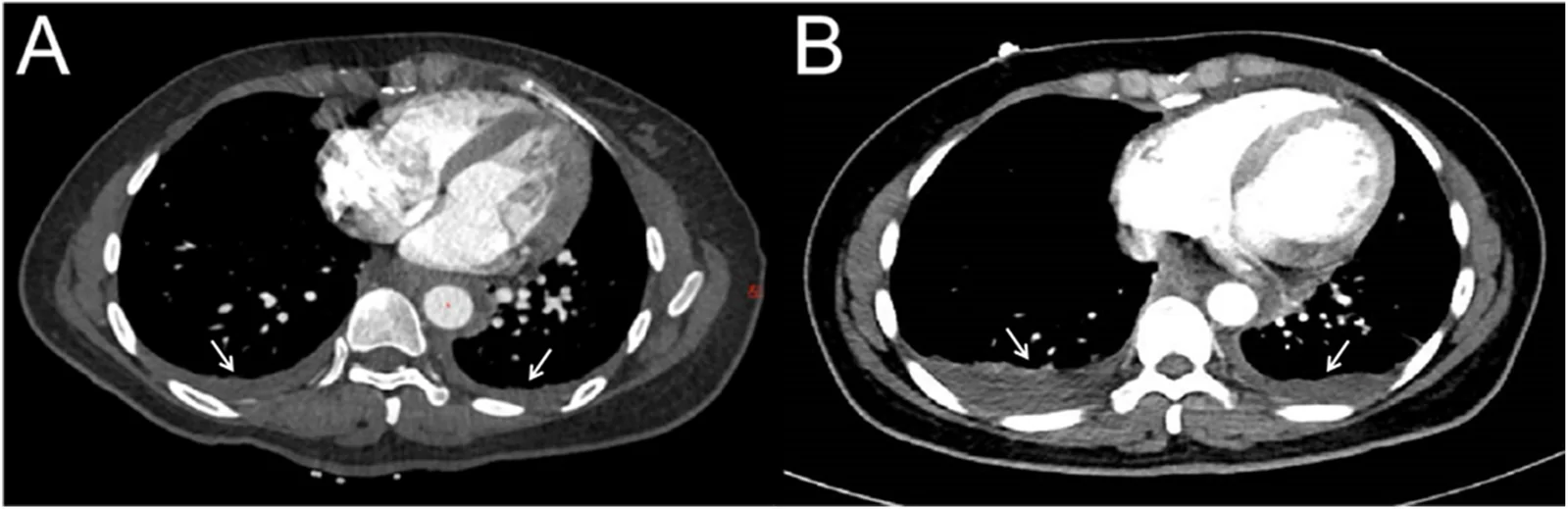
Axial computed tomography (CT) images demonstrating bilateral pleural effusions. **(A)** Initial CT performed on March 15, 2022, showing bilateral pleural effusions (white arrows). **(B)** Repeat CT performed 10 h later demonstrating a marked increase in bilateral pleural effusions (white arrows).

A 38-year-old female was admitted to the emergency department with precordial and shoulder-back pain that persisted for 2 h following a motor vehicle collision. The patient had previously been in good health. On emergency admission on March 15, 2022, the vital signs were as follows: T 36.2℃, P 96 beats/min, R 23 breaths/min, BP 160/102 mmHg. Emergency computed tomography angiography (CTA) of the entire aorta performed on the same day demonstrated a saccular protruding lesion in the aortic arch with a maximum diameter of approximately 1.3 cm, accompanied by discontinuity of the aortic wall (Figure 1). Fluid density shadows were visible in both pleural cavities (Figure 2A). The patient was diagnosed with a thoracic aortic rupture, a thoracic aortic pseudoaneurysm, and bilateral pleural effusion. Laboratory examination revealed the following results: hemoglobin, 112.00 g/L; hematocrit, 32.90%; red blood cell count, 3.70 × 10^12^/L; white blood cell count, 10.38 × 10^9^/L; absolute neutrophil count, 8.36 × 10^9^/L; neutrophil percentage, 80.60%; lymphocyte percentage, 12.00%; D-dimer, 5.99 mg/L; alanine aminotransferase, 80.0 U/L; aspartate aminotransferase, 66.0 U/L; total bilirubin, 23.2 μmol/L; direct bilirubin, 15.6 μmol/L; glucose, 7.03 mmol/L;serum creatinine, 38.3*μ*mol/L; and eGFR, 126.51 mL/min/1.73 m^2^. Following admission, continuous intravenous infusion of sodium nitroprusside and analgesic therapy were administered. Bedside monitoring demonstrated a heart rate fluctuating between 86 and 100 beats/min, blood pressure ranging from approximately 140–190/90–98 mmHg, and oxygen saturation maintained at 97%–99%. However, after 10 h of observation, the patient's pain remained unresolved. Repeat blood routine examination demonstrated a red blood cell count of 3.14 × 10^12^/L, hemoglobin level of 95 g/L, hematocrit of 28.7%, white blood cell count of 10.4 × 10^9^/L, platelet count of 171 × 10^9^/L, absolute neutrophil count of 9.28 × 10^9^/L, and absolute lymphocyte count of 0.54 × 10^9^/L. Repeat thoracic aortic CTA demonstrated no significant change in the size of the saccular aortic lesion, whereas the bilateral pleural effusions had slightly increased compared to the previous examination (Figure 2B). Based on the progression of the clinical condition and repeat laboratory and CTA findings, the patient was considered to have an unstable traumatic disease with a high risk of massive hemorrhage secondary to the rupture of the thoracic aortic pseudoaneurysm. Available treatment options included endovascular repair using a Castor single-branched stent graft, parallel stent techniques such as the chimney technique, and debranching combined with thoracic aortic stent graft implantation. After a detailed communication with the patient's family regarding the technical characteristics, therapeutic objectives, and potential risks of each procedure, informed consent for surgical treatment was obtained. Considering the anatomical characteristics of the lesion, TEVAR using a Castor single-branched stent graft was ultimately selected.

### 2.2 Operation

#### 2.2.1 Preoperative CTA measurement and stent selection

Preoperative CTA measurements demonstrated a proximal sealing zone diameter of approximately 25 mm, a distal sealing zone diameter of approximately 18.8 mm, a left subclavian artery (LSA) diameter of 9 mm, and an iliac access vessel diameter of 8.6 mm. The distance from the pseudoaneurysm entry tear to the LSA origin was <10 mm, and the distance between the origins of the left common carotid artery and the LSA was approximately 7 mm.

The selected Castor single-branched stent-graft had a proximal main-body diameter of 28 mm, a distal main-body diameter of 22 mm, a main-body length of 200 mm, a branch diameter of 10 mm, a branch length of 25 mm, and a 5-mm distance from the proximal end of the main body to the branch opening. The main body of the Castor single-branched stent-graft was oversized by approximately 12% at the proximal sealing zone and 17% at the distal sealing zone relative to the aortic diameter.

#### 2.2.2 Operative procedure

**Figure 3:**
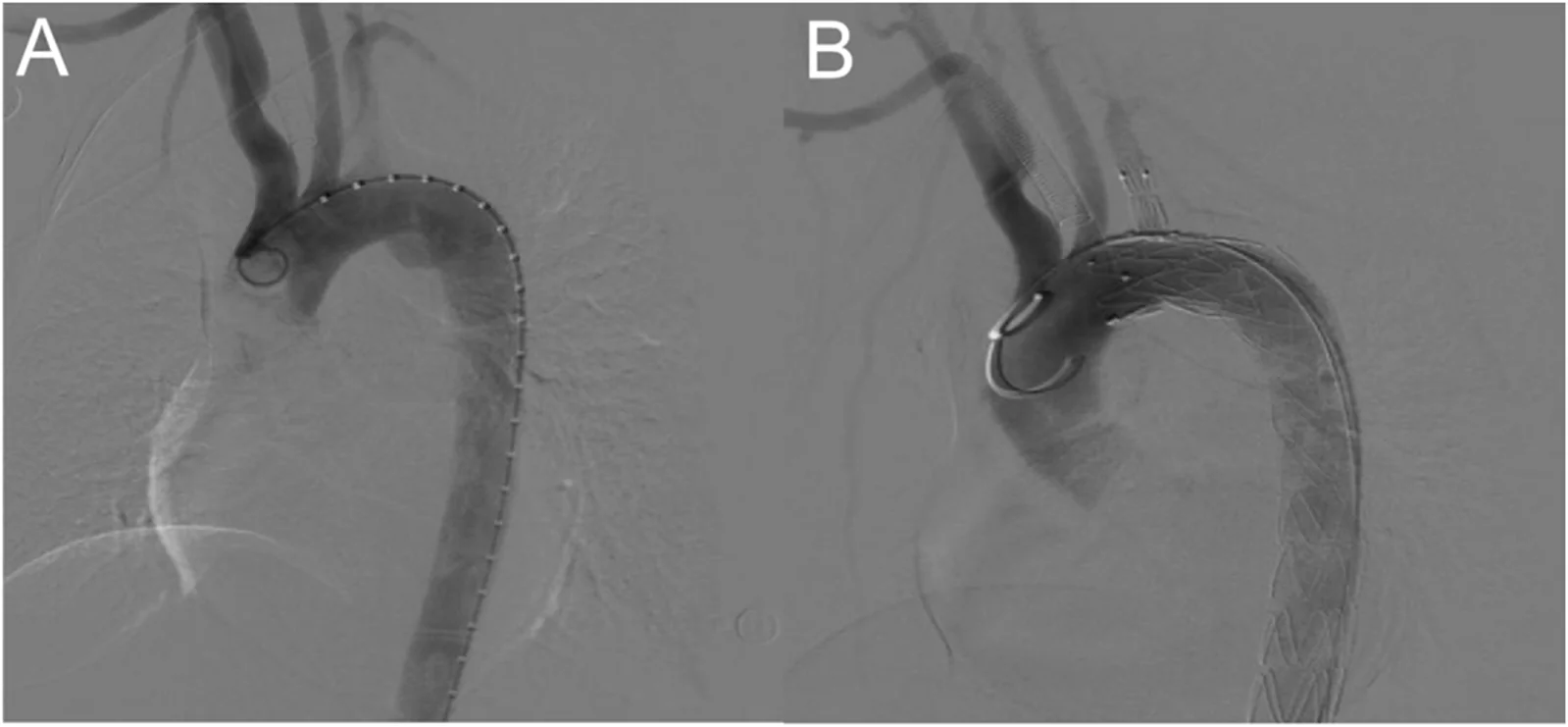
Digital subtraction angiography (DSA) of the thoracic aorta. **(A)** Preoperative DSA demonstrates a saccular aneurysm of the thoracic aorta. **(B)** Postoperative DSA following implantation of a Castor single-branched stent graft system shows appropriate device positioning with unobstructed blood flow in the descending thoracic aorta and its branch vessels.

Following the completion of the preoperative preparation, emergency thoracic endovascular repair using a Castor single-branched stent graft was performed under general anesthesia. The patient was placed in the supine position with endotracheal intubation, and the left upper extremity was abducted after routine skin preparation and sterile draping. 10-F and 6-F vascular sheaths (Cook, USA) were introduced through the right common femoral artery and the left brachial artery, respectively. A 5-F calibrated pigtail catheter (Cordis, USA) was used for angiography, which confirmed the presence of a pseudoaneurysm at the aortic isthmus (Figure 3A). Intraoperative angiographic measurements of the aortic diameter were consistent with the preoperative CTA findings. A working guidewire system was established via the dual-access approach. Under controlled hypotension (systolic blood pressure maintained below 100 mmHg), a Castor single-branched stent graft (MicroPort Endovascular, Shanghai, China) was prepared for deployment. The main body and branch components were sequentially deployed according to the manufacturer's instructions. Final angiography demonstrated complete exclusion of the pseudoaneurysm without residual sac opacification. The branch stent was accurately positioned within the left subclavian artery (LSA), and satisfactory perfusion of the distal thoracic aorta, LSA, and left common carotid artery (LCCA) was confirmed (Figure 3B). The operative time was 80 min, the fluoroscopy time was 30 min, the radiation dose was 136.4 Gy·cm^2^, and the contrast volume was 150 mL. At the completion of the procedure, the vascular access sites and surgical incision were managed routinely and compressed for hemostasis. The patient was successfully extubated without intraoperative complications, remained hemodynamically stable, and was transferred to the general ward, where postoperative intensive care unit (ICU) admission was not required.

### 2.3 Follow-up

**Figure 4:**
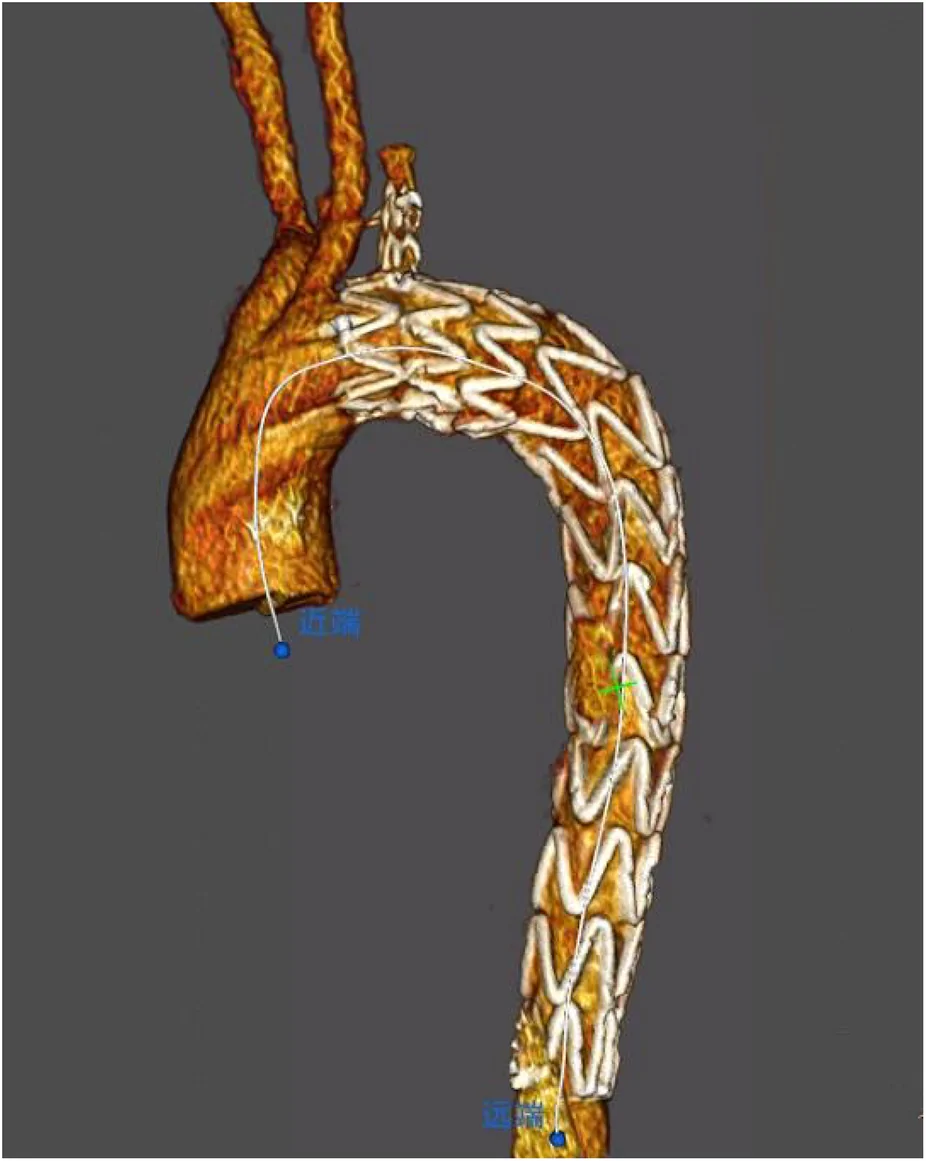
Computed tomography angiography (CTA) of the thoracic aorta at 3-year follow-up shows a well-positioned stent with patent flow in the aortic arch and supra-aortic branches.

The total postoperative hospital stay was 8 days, and no antiplatelet or antithrombotic therapy was prescribed after discharge. Postoperative follow-up was scheduled at 3 months, 6 months, and 1 year after the procedure, and annually thereafter. At each follow-up visit, whole-aorta CTA with delayed-phase imaging was performed to evaluate postoperative recovery and monitor for procedure-related complications. At the 3-year postoperative follow-up, CTA demonstrated a well-positioned stent graft within the aortic arch and descending thoracic aorta with satisfactory aortic remodeling and no evidence of stent migration or endoleak. The LSA remained patent and no opacification of the aneurysmal sac was observed. The bilateral pleural effusions resolved completely (Figure 4).

## 3 Discussion

The clinical presentation of patients with BTAI is often nonspecific at admission. Some patients remain asymptomatic, whereas others may present with chest pain, interscapular pain, dyspnea, hypotension, or impaired consciousness (6, 7). Because chest radiographs have limited sensitivity and specificity for the initial screening of BTAI and are associated with a relatively high false-negative rate, CTA of the aorta is currently regarded as the diagnostic gold standard. CTA provides high sensitivity, specificity, and negative predictive value, enabling rapid identification of the aortic injury site and evaluation of associated thoracic injuries, thereby facilitating timely therapeutic decision-making (6, 8). Transesophageal echocardiography, intravascular ultrasonography, and magnetic resonance imaging may serve as adjunct diagnostic modalities (9). The aortic isthmus is the most commonly involved site in BTAI because it represents the transitional junction between the highly mobile aortic arch proximally and the relatively fixed descending thoracic aorta distally, where the aortic wall is relatively vulnerable and constrained by the surrounding osseous structures. Under sudden deceleration forces, shear stress tends to concentrate at this location, resulting in intimal injury and, in some cases, secondary pseudoaneurysm formation (10, 11). Less commonly, injuries involve the ascending aorta, distal descending thoracic aorta, or abdominal aorta (1). In 2011, the Society for Vascular Surgery (SVS) classified BTAI into four grades according to injury severity: grade I, intimal tear; grade II, intramural hematoma; grade III, pseudoaneurysm; and grade IV, aortic rupture (12). This grading system has important implications in treatment selection and prognostic evaluation. A systematic review and meta-regression analysis including 35 studies and 2,897 patients demonstrated that the aortic-related mortality rate was approximately 1% for grade I–II injuries, but increased to 18% for grade III–IV injuries, indicating a relatively favorable prognosis for low-grade injuries (13). The 2026 SVS guidelines further refine the management strategies for different grades of BTAI. For grade I and II injuries, non-operative management is recommended, and routine TEVAR or open surgical repair is generally not indicated. Patients with grade I injuries usually do not require routine imaging follow-up, whereas repeat CTA within 48–72 h after admission is recommended for grade II injuries to evaluate disease progression (14). Similarly, a retrospective analysis from the Vascular Quality Initiative (VQI) demonstrated that the in-hospital aortic-related mortality rates among conservatively managed patients with grade I and II injuries were only 2.4% and 0.9%, respectively, further supporting conservative management as the preferred strategy for low-grade BTAI (15). For patients undergoing non-operative treatment, close clinical and imaging surveillance should be combined with optimal medical therapy. Anti-impulse therapy is commonly used for hemodynamic management, with systolic blood pressure typically maintained at 90–110 mmHg and heart rate below 100 beats/min, to reduce aortic wall stress and stabilize the lesion. However, in patients with concomitant traumatic brain injury, blood pressure management should prioritize the maintenance of cerebral perfusion; therefore, routine anti-impulse therapy is not recommended (14, 16). For grade III injuries, delayed TEVAR may be considered in hemodynamically stable patients, while associated traumatic injuries are preferentially addressed, whereas early emergency intervention is recommended in the presence of hemodynamic instability secondary to aortic injury. Grade-IV injuries generally require immediate surgical repair (14). Overall, the timing and selection of treatment for BTAI should be individualized according to the patient's clinical manifestations, hemodynamic status, and injury grade (17). In the present case, immediate precordial and shoulder-back pain developed after a motor vehicle accident, and comprehensive evaluation based on CTA findings, laboratory examinations, and dynamic clinical assessment indicated grade III BTAI with poor hemodynamic status and a high risk of rupture, necessitating emergency surgical intervention.

Currently, the primary treatment strategies for BTAI-associated thoracic aortic pseudoaneurysm include open surgical repair, TEVAR, and hybrid procedures, with the optimal therapeutic approach requiring individualized selection according to the patient's clinical condition and anatomical characteristics. Open surgical repair has historically been regarded as the standard treatment for BTAI and has demonstrated reliable efficacy. However, owing to its substantial surgical trauma, high perioperative risk, and prolonged postoperative recovery, a multicenter prospective study reported a mortality rate of up to 23.5%, limiting its application mainly to select patients with anatomy unsuitable for endovascular intervention (18–20). Although hybrid procedures avoid aortic cross clamping and deep hypothermic circulatory arrest, thereby reducing surgical invasiveness to some extent, the associated perioperative morbidity and mortality rates remain considerable (21). In contrast, TEVAR has become the preferred treatment modality for BTAI and is recommended as the first-line strategy in the current guidelines because of its minimally invasive nature, favorable safety profile, and rapid postoperative recovery. In the same study, the mortality rate associated with TEVAR was only 7.5%, which was significantly lower than that associated with open surgical repair. Although complications such as endoleaks and stent migration may still occur after TEVAR, the reported incidence of these complications is generally < 1.2%, indicating an overall acceptable safety profile (19, 22, 23). TEVAR has also demonstrated favorable short- and mid-term outcomes in the management of various thoracic aortic diseases (24). Nevertheless, conventional TEVAR requires an adequate proximal landing zone, typically necessitating a sufficient distance between the lesion and the LSA, as well as a proximal landing zone length greater than 2.0 cm, to achieve stable fixation without coverage of the supra-aortic branch vessels. When the lesion is located adjacent to the LSA origin or the proximal landing zone is insufficient, coverage of the LSA or LCCA may be required to ensure adequate stent apposition and exclusion of the lesion. However, such coverage may increase the risk of complications including stroke, spinal cord ischemia, and upper extremity ischemia, highlighting the clinical importance of preserving LSA perfusion (24–26). Current strategies for LSA revascularization include carotid-subclavian bypass, carotid-subclavian transposition, and branched stent graft implantation (25). Carotid-subclavian bypass effectively restores LSA blood flow by creating an artificial vascular conduit between the LCCA and LSA, although it is associated with greater surgical trauma and graft-related complications (27). Carotid-subclavian transposition reconstructs blood flow through direct anastomosis of the LSA to the common carotid artery, thereby avoiding prosthetic graft implantation; however, the procedure is technically more demanding (28, 29). In comparison, the Castor single-branched stent graft system, as a totally endovascular, minimally invasive technique, enables simultaneous repair of aortic lesions and reconstruction of LSA perfusion while offering the advantages of reduced surgical trauma, less blood loss, faster postoperative recovery, and a lower incidence of complications. Consequently, it has gradually emerged as an important minimally invasive treatment option for complex aortic arch pathologies in recent years (30).

The Castor single-branched stent graft system is the first integrated unibody single-branched aortic stent graft developed in China by MicroPort Medical (Shanghai, China) and is primarily designed for the reconstruction of LSA perfusion during TEVAR for aortic diseases (31). Its integrated branch design enhances the overall stent stability and reduces the risk of component separation while allowing improved conformity to the aortic arch without the need for additional branch stent implantation. Consequently, the device can effectively seal the aortic lesion and exclude the aneurysmal sac while maintaining LSA perfusion, thereby reducing the complications associated with branch vessel occlusion. In addition, the stent graft can be individually customized according to the length of the proximal LSA segment and the morphology of the aortic arch to improve the anatomical compatibility between the device and the patient (32). The device consisted of a polyester graft sutured to a self-expanding nitinol stent framework and was designed without proximal or distal bare stent segments. Its delivery system adopts a dual-sheath configuration in which the aortic main body and branch components are separately constrained, allowing sequential deployment of the main graft and branch section through a trigger wire and traction wire mechanism, thereby improving deployment accuracy (33). Multiple multicenter and single-center studies from Asia have demonstrated that the Castor single-branched stent graft achieves high technical success rates, favorable aortic remodeling, and satisfactory mid-term clinical outcomes, supporting its safety and effectiveness in the treatment of type B aortic dissection (TBAD), although its long-term durability remains to be established through extended follow-up (34–36). More recently, a single-center study from Italy reported similarly favorable early outcomes in a Western population; however, the current European experience remains limited to relatively small cohorts with short follow-up periods (37, 38). In addition, a recent European anatomical feasibility study demonstrated that approximately two-thirds of patients undergoing zone 2 TEVAR were anatomically suitable for implantation of the Castor stent graft. Furthermore, only a limited range of device sizes was required to accommodate the majority of eligible patients, further supporting its potential clinical applicability in zone 2 aortic arch pathologies (39).

In addition to device design, vascular access represents another key determinant of successful TEVAR, particularly in female patients. Previous studies have shown that women generally have smaller iliofemoral artery diameters and vascular anatomical characteristics that differ from those of men, predisposing them to a higher risk of access-related vascular complications during complex endovascular aortic repair requiring large-profile delivery systems (40, 41). In the present case, preoperative CTA demonstrated a right common femoral artery diameter of 8.2 mm and a left common femoral artery diameter of 8.0 mm. Because the maximum outer diameter of the Castor delivery system was 24 F (approximately 8.0 mm), the right common femoral artery, which had the larger vessel diameter, was selected as the access route. Careful device manipulation and continuous assessment of resistance during delivery were performed to minimize the risk of access-related vascular injury.

## Conclusion

The Castor single-branched stent-graft may be a minimally invasive treatment option for patients with blunt thoracic aortic injury-related pseudoaneurysm involving or adjacent to the left subclavian artery, although further studies are required to confirm its safety and efficacy.

## Data Availability

The data presented in this study are available upon request from the corresponding author(s).
